# The effect of screening and treatment of *Ureaplasma urealyticum* infection on semen parameters in asymptomatic leukocytospermia: a case–control study

**DOI:** 10.1186/s12894-020-00742-y

**Published:** 2020-10-22

**Authors:** Qi-Feng Zhang, Yu-Ji Zhang, Sheng Wang, Yu Wei, Feng Li, Ke-Jian Feng

**Affiliations:** 1 Department of Andrology, Guilin People’s Hospital, Guilin, 541002 China; 2 Department of Medical Record Management, The First Affiliated Hospital of Hainan Medical University, Haikou, 571137 China; 3 Department of Urology, Guilin People’s Hospital, Guilin, 541002 China

**Keywords:** Leukocytospermia, *Ureaplasma urealyticum*, Semen parameters, Doxycycline

## Abstract

**Background:**

*Ureaplasma urealyticum* (UU) infection, as well as asymptomatic leukocytospermia, whether it has effect on semen parameters and whether it needs screening and treatment is still a confusing and controversial topic for clinicians.

**Methods:**

Among 1530 adult males who visited Guilin People's Hospital due to infertility, 295 were diagnosed with asymptomatic leukocytospermia, and 95 were further screened for UU-positive. 81 UU-positive asymptomatic leukocytospermia patients received 7-day or 14-day treatment plan with doxycycline, and 70 cases were cured. The semen parameters of non-leukocytospermia, leukocytospermia, UU-positive leukocytospermia and UU-negative leukocytospermia groups were compared, and the differences between the two treatment plans and the semen parameters before UU treatment and 1 month after UU-cured were compared.

**Results:**

Compared with non-leukocytospermia patients, the sperm concentration, progressive motility (PR), and normal morphology of patients with leukocytospermia decreased, while those with UU-positive leukocytospermia performed more significantly. The PR, total motility, and normal morphology of UU-positive leukocytospermia patients were significantly lower than those of UU-negative leukocytospermia patients (all *p* < 0.001). The UU cure rates of the 7-day and 14-day treatment plan with doxycycline was 84.62% and 89.66% (*p* = 0.738), respectively, and the sperm concentration, PR, total motility, and normal morphology of the cured UU-positive leukocytospermia patients were all increased after 1 month (*p* = 0.001, *p* = 0.022, *p* = 0.004 and *p* = 0.008, respectively).

**Conclusions:**

It is significant to screen and treat UU infection in asymptomatic leukocytospermia for improving sperm quality. Where appropriate, the 7-day treatment plan with doxycycline may be a good choice.

## Background

Leukocytospermia is an abnormal laboratory finding defined by the World Health Organization as the presence of 1 × 10^6^ seminal leukocytes per mL in human ejaculate [1], It is generally accepted that leukocytospermia may indicate infection or inflammation of the male urogenital and sex glands tract [2, 3], and it is a poor marker for either bacteriospermia or impaired semen quality [4], which has a negative impact on spermatogenesis or maturation, and is related to the decrease of semen parameters such as sperm concentration, motility, normal morphology and DNA fragmentation index [5, 6]. A prospective, controlled, blinded study suggests that leukocytospermia has a significant effect on sperm dynamic motility patterns, DNA and chromosome integrity in infertile men, and the pregnancy rate of patients cured with antibiotics is significantly higher than that of patients with persistent leukocytospermia [7]. Studies showed that antibiotics might improve the rate of resolution of leukocytospermia, the bacteriologic cure rate, and even improve sperm parameters to increase pregnancy rate [8]. A meta-analysis of 12 studies showed that using broad spectrum antibiotics in the treatment of patients with leukocytospermia might be useful in improving sperm concentration, motility, and morphology [9]. But a number of studies have shown that there is no correlation between elevated levels of semen leukocytes and bacteriospermia [10], and evidence from an RCT study also suggests that antibiotic therapy is not beneficial for the treatment of asymptomatic leucocytospermia [11], even in infertile men, leukocytospermia does not affect the success rate of IVF or ICSI [12]. Although a large number of studies have proved the relationship between semen leukocytosis and bacterial infection, sperm quality, and even the effectiveness of antibiotic treatment, the conflicting results are not convincing, the association of leukocytospermia with semen quality or male fertility and the significance of antibiotic therapy are still under debate.

Similarly, asymptomatic carrying of *Ureaplasma urealyticum* (UU) and *Mycoplasma hominis* (MH) is common, most people will not develop into diseases, and there is not enough evidence to prove that it is beneficial to detect and treat these infections, so it is not recommended to carry out routine detection and treatment for asymptomatic or symptomatic men and women [13, 14]. On the other hand, some studies suggest that the presence of UU and MH is related to the abnormal sperm parameters [15], sperm vitality and Progressive motility of UU infected men were significantly lower than those of uninfected men [16], and the mean sperm concentration was also lower [17]. The relationship between UU infection and sperm quality or male fertility is also debated.

Therefore, we designed this study to find more evidence of the effect of asymptomatic leukocytospermia and UU infection on sperm quality and whether antibiotic therapy is beneficial.

## Methods

We retrospectively analyzed adult males who visited Guilin People's Hospital due to infertility and performed routine semen analysis and UU screening and treatment for patients with leukocytospermia from April 2017 to December 2019. After excluded patients with systemic diseases, cryptorchidism, chromosome abnormalities, varicocele, male azoospermia, bacteriospermia, as well as patients who drank excessively, smoked heavily, had symptoms of genitourinary tract infection or a history of antibiotic use in recent 4 weeks, 1530 patients and medical records were finally collected. Among them, 295 cases were diagnosed with leukocytospermia, 95 cases were further screened for UU-positive, 81 cases were treated with doxycycline, and 14 cases were treated with other sensitive antibiotics due to antibiotic sensitivity tests showing doxycycline resistance or intermediate sensitivity, or for other reasons. The semen parameters of non-leukocytospermia, leukocytospermia, UU-positive leukocytospermia and UU-negative leukocytospermia groups were compared, and the differences between two treatment plans with doxycycline and the semen parameters before UU treatment and 1 month after UU-cured were compared. The study was approved by the ethical review committee.

Semen samples were collected in sterile containers by masturbation after 2–7 days of abstinence. Sperm Quality Analyzer (SQA-V, Medical Electronic Systems Co., Ltd.) was used for the semen analysis within 60 min after ejaculation and liquefaction, and the semen volume, pH value, sperm concentration, progressive motility (PR), total motion (PR + NP) (NP, Non-progressive motility) and normal morphology were measured by the World Health Organization (WHO, 5th Edition). The seminal leukocytes was detected using the peroxidase method and leukocytospermia was diagnosed by seminal peroxidase-positive leukocyte concentration not less than 1 × 10^6^ per mL. Culture appraisal of UU/MH was carried out with urethral swab samples by the modified broth dilution testing methods (Kit provided by Zhuhai Langfeng Biotech Co., Ltd.), and reexamined by the same method at least one week after treatment. Antibiotic sensitivity test results were obtained at high and low concentrations for 12 antibiotics: Cyclolipoerythromycin, Doxycycline, Josamycin, Thiamphenicol, Clarithromycin, Erythromycin, Ciprofloxacin, Roxithromycin, Levofloxacin, Minocycline, Azithromycin and Gatifloxacin. The susceptibility of bacteria to each antibiotic was graded as either susceptible, intermediate or resistant. For UU-positive leukocytospermia patients with doxycycline susceptible, no history of allergies, and contraindications, doxycycline was preferred, and 7-day or 14-day treatment plan (doxycycline 100 mg twice daily for 7 days or 14 days, double the first dose) options were selected according to the patient's wishes.

Data analysis was performed using IBM SPSS Statistics for Windows, Version 24 (IBM Corp., Armonk, New York, United States). Compliance of variables with a normal distribution was analyzed with the Kolmogorov–Smirnov test. Continuous variables were presented as means with standard deviations (mean ± SD) or medians (25–75%). Categorical variables were represented as numbers with percentages. Continuous data were analyzed by Independent-samples t-test or Paired-samples t-test for normally distributed variables and Mann–Whitney U test or Wilcoxon Signed-Ranks Test for non-normally distributed variables. Categorical data were analyzed using Pearson's chi-square test or Fisher’s exact test. Statistical significance was defined as *p* < 0.05.

## Results

According to the detection of seminal leukocytes, 1235 of 1530 infertile patients were diagnosed with non-leukocytospermia and 295 with leukocytospermia. The sperm concentration, PR and normal morphology of patients with leukocytospermia were lower than those of the patients with Non-leukocytospermia (*p* < 0.001, *p* = 0.017 and *p* = 0.019, respectively). According to the culture appraisal, 95 patients with leukocytospermia were positive for UU (8 cases of them were UU and MH positive at the same time, there was no single MH positive), while 200 patients were negative. Compared with Non-leukocytospermia patients, the sperm concentration and total motility of UU-negative leukocytospermia patients decreased significantly (*p* < 0.001 and *p* = 0.022, respectively), while the sperm concentration, PR, total motility and normal morphology of UU-positive leukocytospermia patients decreased significantly (all *p* < 0.001). Compared with UU-negative and UU-positive leukocytospermia group, The PR, total motility and normal morphology of UU-positive leukocytospermia patients were significantly lower than those of UU-negative leukocytospermia patients (all *p* < 0.001). (Table 1).

**Table 1:** General information and comparison of Non-leukocytospermia group, leukocytospermia group, UU-negative leukocytospermia group and UU-positive leukocytospermia group

	Non-leukocytospermia group	Leukocytospermia group	UU-negative leukocytospermia group	UU-positive leukocytospermia group	^1^*p *value^a^	^2^*p *value^a^	^3^*p *value^a^	^4^*p *value^a^
n	1235	295	200	95				
Age (years)	33.32 (29.00–37.00)	33.20 (29.00–37.00)	33.39 (29.00–37.00)	32.80 (29.00–36.00)	0.530	0.408	0.952	0.543
BMI (kg/m^2^)	21.24 (19.94–22.39)	21.29 (20.17–22.38)	21.32 (20.34–22.38)	21.22 (19.66–22.39)	0.544	0.930	0.201	0.277
Semen volume (mL)	3.52 (2.50–4.50)	3.42 (2.50–4.50)	3.47 (2.50–4.50)	3.31 (2.50–4.50)	0.275	0.658	0.163	0.326
PH	7.66 (7.30–8.00)	7.69 (7.30–8.10)	7.71 (7.40–8.00)	7.63 ± 0.63	0.253	0.121	0.859	0.330
Sperm concentration (× 10^6^/mL)	82.13 (43.00–113.8.80)	65.55 (33.60–83.30)	66.29 (38.88–82.45)	63.99 (24.4–92.00)	< 0.001	< 0.001	< 0.001	0.228
PR (%)	36.02 (27.00–47.00)	31.80 (9.00–49.00)	35.23 (19.25–53.00)	24.58 (0–39.00)	0.017	0.514	< 0.001	< 0.001
Total motility, PR + NP (%)	47.55 (40.00–59.00)	43.35 (24.00–64.00)	47.64 (34.50–66.00)	34.33 (4.00–55.00)	0.510	0.022	< 0.001	< 0.001
Normal morphology (%)	8.57 (5.00–11.00)	7.81 (3.00–8.00)	8.70 (4.00–13.00)	5.93 (0–9.00)	0.019	0.492	< 0.001	< 0.001

*UU* *Ureaplasma urealyticum*, *BMI* body mass index, *PR* progressive motility, *NP* non-progressive motility

^a^Mann–Whitney U-test

^1^*p *value (Comparison between non-leukocytospermia group and leukocytospermia group)

^2^*p *value (Comparison between non-leukocytospermia group and UU-negative leukocytospermia group)

^3^*p *value (Comparison between non-leukocytospermia group and UU-positive leukocytospermia group)

^4^*p *value (Comparison between UU-negative leukocytospermia group and UU-positive leukocytospermia group)

**Fig. 1:**
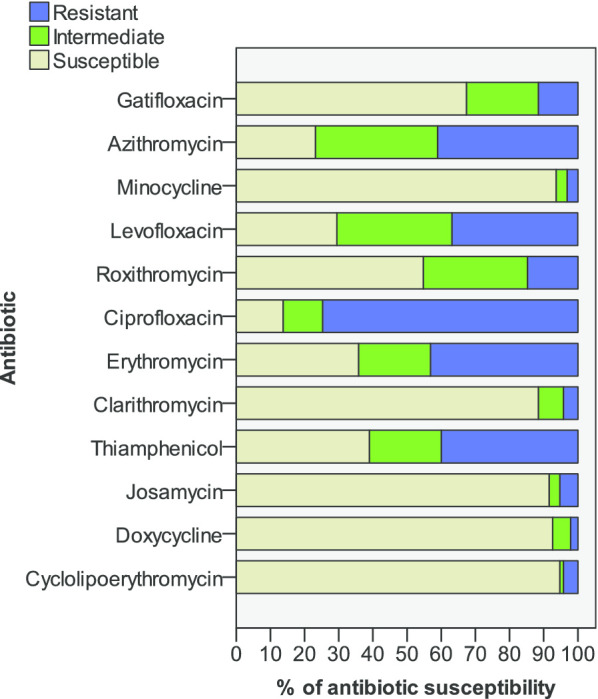
Susceptibility of *Ureaplasma urealyticum* to 12 different antibiotics

Antibiotic sensitivity test showed that the susceptible rate of Cyclolipoerythromycin was 94.74%, Minocycline was 93.68%, and doxycycline was 92.63% (Fig. 1), As doxycycline is inexpensive and easily available, we usually recommend doxycycline for patients who are susceptible to doxycycline and have no history of allergies or contraindications, and choose 7-day or 14-day treatment plan according to the patient's wishes. As a result, 14 UU-positive patients were treated with other sensitive antibiotics due to antibiotic sensitivity tests showing doxycycline resistance or intermediate sensitivity, or for other reasons, and 81 UU-positive patients received doxycycline treatment. We collected 52 patients with a 7-day treatment plan and 29 patients with a 14 day treatment plan with doxycycline, the cure rates were 84.62% and 89.66% respectively, and there was no significant difference between the two treatment plans (*p* = 0.738). (Table 2). At the same time, we also found that the two treatment plans have no significant difference in semen volume, PH, sperm concentration, PR, total motility and normal morphology for UU cured patients (*p* = 0.490, *p* = 0.529, *p* = 0.995, *p* = 0.592, *p* = 0.486 and *p* = 0.293, respectively). (Table 3).

**Table 2:** Comparison of UU cure rates between the two treatment plans

	n	UU cured case (%)	UU uncured case (%)	*p *value^a^
7-day treatment plan	52	44 (84.62)	8 (15.38)	0.738
14-day treatment plan	29	26 (89.66)	3 (10.34)	

*UU* *Ureaplasma urealyticum*

^a^Fisher's exact test

**Table 3:** Comparison of semen parameters of UU-positive leukocytospermia patients before treatment and 1 month after UU Cured

	Before treatment		*p *value	1 month after UU Cured		*p *value
	7-day treatment plan	14-day treatment plan		7-day treatment plan	14-day treatment plan	
n	44	26		44	26	
Semen volume (mL)	3.12 (2.13–4.00)	3.34 ± 1.67	0.659^a^	3.15 (2.50–3.38)	3.44 (2.50–4.13)	0.490^a^
PH	7.60 ± 0.66	7.67 ± 0.61	0.693^b^	7.62 ± 0.65	7.72 ± 0.49	0.529^b^
Sperm concentration (× 10^6^/mL)	65.13 (28.40–99.38)	59.96 ± 45.44	0.738^a^	70.25 (34.60–87.28)	68.04 ± 44.94	0.995^a^
PR (%)	23.20 (0.25–35.75)	25.23 (2.5–40.75)	0.359^a^	25.43 ± 18.07	27.82 ± 17.85	0.592^b^
Total motility, PR + NP (%)	32.91 (5.00–50.75)	36.42 (12.5–59.00)	0.274^a^	36.72 ± 20.27	40.21 ± 19.82	0.486^b^
Normal morphology (%)	5.52 (0.50–8.00)	5.96 (1.50–9.25)	0.401^a^	6.09 (2.25–9.00)	7.31 ± 4.41	0.293^a^

*UU* *Ureaplasma urealyticum*, *PR* progressive motility, *NP* non-progressive motility

^a^Mann–Whitney U-test

^b^Independent-samples t-test

A further study of 70 UU-positive leukocytospermia patients before and after treatment showed that the sperm concentration, PR, total motility, and normal morphology of the UU cured patients were all increased after 1 month after UU-cured (*p* = 0.001, *p* = 0.022, *p* = 0.004 and *p* = 0.008, respectively), but there was no significant difference in semen volume and pH value (*p* = 0.324 and *p* = 0.663, respectively). (Table 4).

**Table 4:** Comparison of semen parameters of UU-positive leukocytospermia patients before treatment and 1 month after UU cured

	Before treatment	1 month after UU Cured	*p *value^a^
Semen volume (mL)	3.20 (2.08–4.00)	3.26 (2.50–4.00)	0.324
PH	7.63 (7.15–8.20)	7.66 ± 0.59	0.663
Sperm concentration (× 10^6^/mL)	63.21 (24.00–95.88)	69.43 (35.03–88.73)	0.001
PR(%)	23.96 (1.00–39.00)	26.32 (9.75–41.25)	0.022
Total motility, PR + NP (%)	34.21 (7.25–53.25)	38.02 ± 20.03	0.004
Normal morphology (%)	5.69 (1.5–9.00)	6.54 (3.00–10.00)	0.008

*UU* *Ureaplasma urealyticum*, *PR* progressive motility, *NP* non-progressive motility

^a^Wilcoxon signed-ranks test

## Discussion

Leukocytospermia or pyospermia is still a confusing and controversial topic for clinicians. Although it has been proposed by the World Health Organization as an indicator of reproductive tract infection and inflammation, the evidence is not convincing. The frequency of leukocytospermia among infertile males is quite wide, and the reliable incidence is about 30% [18, 19]. Some pathophysiological views recognize that bacteria and infiltrating leukocytes, two major inflammatory mediators, directly or indirectly affect human sperm function [20], However, most leukocytospermia is not caused by bacterial infection or bacteriospermia, or there is no evidence of microbial infection, this may be because the invasion of male reproductive tract by microorganisms may be divided into three different stages. In the first stage, bacterial infection can be induced by microorganisms, and pathological bacterial strains can be observed in semen, without attracting a large number of white blood cells; in the second stage, activated white blood cells appear in ejaculation; in the third stage, bacteria are removed, usually with isolated leukocytospermia as the representative [21]. Bacteria are mainly involved in the intrinsic and mitochondrial dependent apoptosis cell death, while oxidative stress may play a role in reducing the conventional sperm parameters in leukocytospermia, and the combined effect of bacteria and leukocytes accelerated the apoptosis and necrosis of sperm [22]. The increased seminal leukocytes may mediate reactive oxygen species (ROS) production by direct cell–cell contact or soluble products released by leukocytes [23]. A Systematic Review and Meta-Analysis of Case–Control Studies indicated that patients with leukocytospermia showed lower sperm concentration and lower progressive motility compared to men without leukocytospermia, however, the significant differences disappeared, along with the large inter-study heterogeneity, when analyses were restricted to studies clearly reporting the inclusion of men without clinical evidence of seminal tract infection [24]. It seems that the clinical evidence of seminal tract infection is the key to sperm quality decline, and clinical studies have also found that compared with some pathogenic bacterial strains, the direct contact between conditioned pathogenic bacteria and sperm may play a greater role in promoting apoptosis [25].

*Ureaplasma urealyticum* (UU), usually considered as a conditional pathogen, belongs to the genus Ureaplasma and the family Mycoplasmataceae in the order Mycoplasmatales. A meta-analysis found that UU, but not undifferentiated Ureaplasma infections or U. parvum, has been shown to be the pathogen of nongonococcal urethritis (NGU) [26]. Moreover, UU is easy to be implanted in urethra, but not necessarily cause urethritis [27], quite a few patients with these infections are not aware of their infections because they may be asymptomatic, even whether it is pathogenic and the impact on fertility is still uncertain. The incidence of UU was reported inconsistently, a study of 19,098 infertile men and 3368 fertile men found that 10.22% and 3.65% of UU infertile and fertile men's urethra specimens, and 3.16% and 0.89% of MH specimens, UU infection will significantly affect sperm quality, and there is a significant relationship between UU infection and male infertility [28]. A systematic review and meta-analysis found that there was a significant relationship between UU and *Mycoplasma hominis* with male infertility, and compared with the global average, the UU-positive rate was significantly higher in both the infertility group and the control group in China, while the positive rate was significantly lower in *Mycoplasma hominis* [29]. Similarly, UU infection can directly affect sperm, cause leukocyte-mediated inflammatory response, and affect sperm by producing ROS [30].

Oxidative stress (OS) is considered to be the pathological molecular mechanism of most clinical, environmental and lifestyle factors that lead to male infertility. OS occurs when the physiological balance of oxidants and reducers in the system is reduced by excessive ROS or antioxidant levels [31], including some internal and external factors, such as inflammation and infection, varicocele, smoking, drinking, obesity, exposure to radiation and chemotherapy [32], and even male reproductive hormone disorders [33]. In the process of spermatogenesis, reactive oxygen species are very harmful, and the content of unsaturated fatty acids in sperm membrane is easily affected by oxidative stress, and these lipids can be oxidized through a series of chain reactions to release potential toxicity and mutagenic aldehydes and alkenals [34–36], finally, excessive ROS has a pathological impact on spermatogenesis, resulting in the decrease of sperm concentration, motility and fertilization rate [37]. A large number of clinical studies have shown that leukocytospermia-induced sperm damage may be due to high levels of leukocyte-derived ROS and inflammatory mediators, Such as toll-like receptors 2 and 4 (TLR-2/4), cyclooxygenase-2 (COX-2), nuclear factor erythryoid-2 related factor (Nrf-2), interleukin-6 (IL-6) and tumor necrosis factor-alpha (TNF-α), etc. [38, 39], even low levels of leukocytospermia have seminal oxidative stress [40]. It is not certain that the effect of leukocytospermia or UU infection on sperm exists, but it seems to be explainable from the current mechanism research. However, on this issue, clinicians need more clinical evidence and feasible clinical solutions.

In our observation, there was no significant change in the semen volume and pH in all groups. Patients with leukocytospermia have lower sperm concentration, PR and normal morphology than patients with non-leukocytospermia. However, when we exclude UU-positive leukocytospermia patients and only consider UU-negative leukocytospermia patients, although there was a difference in the total motility, the difference between the PR and normal morphology disappeared, but only consider the UU-positive leukocytospermia patients, the sperm concentration, PR, total motility and normal morphology were significantly lower than the patients with non-leukocytospermia. At the same time, compared with UU-negative leukocytospermia patients, the PR, total motor ability and normal morphology of UU-positive leukocytospermia patients were significantly lower. These results seem to confirm that leukocytospermia has a definite effect on semen parameters, at least in terms of sperm concentration. Perhaps the leukocytes in semen itself is a factor that affects semen parameters, of course, these need more extensive research to confirm. However, the effect on PR and normal morphology, like many controversial studies on leukocytospermia, is not convincing. Our research showed that UU-negative leukocytospermia was not like leukocytospermia in terms of PR and normal morphology, which was no significantly different from non-leukocytospermia. Such results indicate that the effect of UU-negative leucospermia on sperm quality is rather limited. However, when patients with leukocytospermia are infected with UU at the same time, the effect on sperm quality is more obvious and serious, which can involve multiple aspects of semen parameters such as PR, total motor ability and normal morphological. This seems to suggest that infectious factors may be an important factor in the decline of sperm quality in asymptomatic leukocytospermia, and UU infection is a common infectious factor in asymptomatic leukocytospermia. Therefore, screening for UU infection in asymptomatic leukocytospermia is meaningful.

According to the antibiotic sensitivity test of UU, the top three susceptible antibiotics were Cyclolipoerythromycin, Minocycline and Doxycycline, and because doxycycline is inexpensive and easy to obtain, we prefer to choose doxycycline in clinic. According to the results of the 7-day and the 14-day treatment plan, there is no significant difference in cure rates, and there is no significant difference in the effect of the two plans on the sperm quality of UU cured leukocytospermia patients. If patients are susceptible to doxycycline and have no history of allergies and contraindications, we think that the doxycycline 7-day treatment plan is a good choice and this antibiotic treatment is meaningful. From our results, the semen parameters such as sperm concentration, PR, total motility, and normal morphology of UU-positive leukocytospermia patients were significantly improved 1 month after UU was cured. Unfortunately, some patients also received antioxidant and other treatments after our observation period, and no correct pregnancy rate was observed. However, our research shows that screening and treating UU in asymptomatic leukocytospermia is meaningful, not only can improve sperm quality but also save a lot of resources and costs, reduce the use of antibiotics, which seems to be an efficient diagnosis and treatment program.

This study has some limitations. Since more pathogens such as chlamydia or mycoplasma have been tested only in symptomatic leukocytespermia rather than asymptomatic leukocytospermia, this may miss some pathogens. In addition, some patients received additional treatments later, and we were unable to follow up with the UU-cured patients for longer periods to obtain more data.

## Conclusion

Asymptomatic leukocytospermia may have some effect on sperm parameters, which seem to be mainly manifested in sperm concentration. When patients with asymptomatic leukocytospermia are infected with UU at the same time, the effect on sperm quality is more obvious and serious, which can involve multiple aspects of semen parameters such as PR, total motor ability and normal morphological. It is significant to screen and treat UU infection in asymptomatic leukocytospermia for improving sperm quality. Where appropriate, we recommend the 7-day treatment plan with doxycycline.

## Data Availability

The datasets used and/or analysed during the current study are available from the corresponding author on reasonable request.

## Abbreviations

UU: *Ureaplasma urealyticum*
MH: *Mycoplasma hominis*
BMI: Body mass index
PR: Progressive motility
NP: Non-progressive motility
